# Supplementary material for: Detection and Genotypic Analysis of Anaplasma bovis and A. phagocytophilum in Horse Blood and Lung Tissue
Source: Int J Mol Sci. 2023 Feb 7;24(4):3239. doi: 10.3390/ijms24043239 (PMC9966372; doi:10.3390/ijms24043239)
Supplement: Supplementary file 1 [file ijms-24-03239-s001.zip › ijms-2155802-supplementary.pdf]

Supplementary Table S1: Primers used for the detection of *Anaplasma* spp. and housekeeping gene from horses in the present study.

Organism	Gene	Primer	Sequence 5′ to 3′	Size (bp)	Amplification condition	Reference
Anaplasma spp.	16S rRNA	EE1	TCCTGGCTCAGAACGAACGCTGGCGGC	1433	94°C/5 min; 35 cycles: 94°C/30 s, 50°C/30 s, 72°C/60 s; 72°C/7 min	[8, 9]
		EE2	AGTCACTGACCCAACCTTAAATGGCTG			
		EE3	GTCGAACGGATTATTCTTTATAGCTTGC	924–926	94°C/5 min; 35 cycles: 94°C/30 s, 50°C/30 s, 72°C/60 s; 72°C/7 min	
		EE4	CCCTTCCGTTAAGAAGGATCTAATCTCC			
Anaplasma phagocytophilum	16S rRNA	EE1	TCCTGGCTCAGAACGAACGCTGGCGGC	1433	94°C/5 min; 35 cycles: 94°C/30 s, 50°C/30 s, 72°C/60 s; 72°C/7 min	[9]
		EE2	AGTCACTGACCCAACCTTAAATGGCTG			
		SSAP2f	GCTGAATGTGGGGATAATTTAT	641	94°C/5 min; 40 cycles: 94°C/30 s, 52°C/30 s, 72°C/60 s; 72°C/5 min	
		SSAP2r	ATGGCTGCTTCCTTTCGGTTA			
Anaplasma bovis	16S rRNA	EE1	TCCTGGCTCAGAACGAACGCTGGCGGC	1433	94°C/5 min; 35 cycles: 94°C/30 s, 50°C/30 s, 72°C/60 s; 72°C/7 min	[9]
		EE2	AGTCACTGACCCAACCTTAAATGGCTG			
		AB1f	CTCGTAGCTTGCTATGAGAAC	551	94°C/5 min; 40 cycles: 94°C/60 s, 55°C/60 s, 72°C/60 s; 72°C/5 min	
		AB1r	TCTCCCGGACTCCAGTCTG			
Housekeeping gene	18S rRNA	F	ATGCGGCGGCGTTATTCC	204	95°C/5 min; 35 cycles: 95°C/20 s, 60°C/30 s, 68°C/60 s; 68°C/5 min	[19]
		R	GCTATCAATCTGTCAATCCTGTCC			
